# Malocclusion Complexity in Patients with Myofascial Pain with or without Mouth-Opening Limitation: A Case-Control Study

**DOI:** 10.1155/2022/3594246

**Published:** 2022-06-08

**Authors:** Iván Daniel Zúñiga-Herrera, José Rubén Herrera-Atoche, Fernando Javier Aguilar-Pérez, Mauricio Escoffié-Ramírez

**Affiliations:** Department of Orthodontics, School of Dentistry, Universidad Autónoma de Yucatán, Yucatán, Mexico

## Abstract

**Background:**

This study is aimed at determining the association between myofascial pain with or without mouth-opening limitation and malocclusion complexity.

**Methods:**

A prospective, cross-sectional, case-control study was conducted. The Research Diagnostic Criteria were used to evaluate the presence of myofascial pain, chronic pain, and depression. The Index of Complexity, Outcome, and Need (ICON) was applied to quantify malocclusion complexity. A total of 96 patients with myofascial pain were grouped into two: subjects without mouth-opening limitation (*n* = 76, group A) and subjects with mouth-opening limitation (group B, *n* = 20). Both groups were compared with 231 controls (group C). A Chi-squared test and a multinomial logistic regression (*p* ≤ 0.05) were used to identify associations between the variables.

**Results:**

Statistically significant associations were found between myofascial pain and the variables gender, malocclusion complexity, and depression (*p* ≤ 0.05). Age was not significantly associated (*p* = 0.327). Concerning malocclusion complexity, 77.9% of the controls were distributed in the first three ICON levels; however, 76.5% of group A subjects and 90% of group B were in the last three (*p* < 0.001). The multinomial logistic regression showed a significant association between malocclusion complexity in group A (*p* < 0.05) and an association between depression and group B (*p* < 0.05). Group B had the highest grades of chronic pain.

**Conclusions:**

Females had greater risk of myofascial pain without mouth-opening limitation. As the complexity of the malocclusion increases, so do the odds of presenting myofascial pain without mouth-opening limitation. Myofascial pain with mouth-opening limitation frequently coexists with depression and chronic pain.

## 1. Background

Pain related to temporomandibular disorders (TMDs) is the principal cause of nondental pain in the orofacial region [1], and among TMD patients, myofascial pain is the most prevalent of these disorders [2]. In some cases, the pain can be acute, which temporarily incapacitates the patient. Patients usually seek dental attention when experiencing this type of pain. When the pain is associated with some other regions, it may be difficult to determine its cause due to diffuse features [3, 4].

TMD comprises a varied group of pathologies (muscle disorders, disc displacements, and arthritic disorders) [5] with multiple causes [6]. Despite gender (females are reported to be at more risk) [7] and trauma [8], other suggested etiological factors seem controversial. For example, it has been proven that TMD prevalence has two age peaks. The first occurs because of a high prevalence of disc displacement disorders (around 30 to 35 years of age), and the second occurs due to arthritic disorders (approximately 50 to 55 years of age). However, muscular disorders are usually mixed with disc displacement or arthritic disorders, making it difficult to establish a peak age for them [9].

Clinicians consider bruxism to be an etiological factor for TMD. However, this relationship is controversial mainly because of the different methods used to diagnose bruxism. Methods to diagnose bruxism, such as self-reporting or clinical examination, are prone to potential bias, and some authors have suggested the necessity to improve the methodological approach [10].

Another group of variables is the psychological factors [11]. Depression, anxiety, and somatization all have been related to TMD [12–14], but it is unclear if it is a cause-effect relationship [14]. In the case of myofascial pain, some authors believe that pain could be an expression of somatization. Therefore, they advise caution before drawing any conclusions [14]. This situation is even more complex when myofascial pain is accompanied by mouth-opening limitation. This limitation of the jaw movement is a splinting reaction to the myofascial pain, and it allows for the healing of the muscles [15]. It is known that jaw disability and myofascial pain are associated with depression [13, 14] and diminished quality of life [16]. Many times, myofascial pain is accompanied by chronic pain [14], resulting from physical disability, poor sleep quality, and deterioration in cognitive abilities [17], which can have severe consequences on patients' daily lives.

Finally, some morphological traits concerning TMD have been studied. In this category, presence of malocclusion can be included. Literature shows that at best, certain malocclusion traits are weakly associated with TMD: (1) increased overjet, (2) open bite, (3) Angle Class III malocclusion, and (4) crossbites are some examples [18]. A systematic review concluded that none of those malocclusion traits are etiological factors for TMD [19]. However, malocclusion is a complex condition in which all of those traits could coexist in the same individual. When studied together, results showed that the increment of malocclusion complexity also increases the odds of presenting TMD [20, 21]. This phenomenon has been attributed to the negative impact that malocclusion has on the quality of life [22, 23] and effects on psychological well-being. Given this evidence, the combination of myofascial pain, malocclusion, and psychological variables previously described may negatively impact a subject's well-being. On the other hand, evidence shows that treating myofascial pain [24] and the correction of malocclusion [25] both improve an individual's quality of life. In this regard, the authors of this paper did not find studies addressing the relationship between myofascial pain and malocclusion complexity.

This study is aimed at determining the possible association between myofascial pain with or without mouth-opening limitation and malocclusion complexity. The study design also included age, gender, bruxism, depression, and chronic pain as variables.

## 2. Material and Methods

An observational, prospective, cross-sectional, case-control study was conducted after approval from a research ethics committee was obtained (CIRB-2017-004). Subjects were asked to sign a voluntary consent form and were fully informed about the study's objectives and procedures. Sample size estimation was based on Breslow's protocol for case-control studies [26]. Sample estimation was conducted considering an odds ratio (OR) of 2 for myofascial pain associated with malocclusion traits. Considering that 61% of the cases presented at least one of the traits [27] and two controls were matched with one case, a sample size of 90 by 179 provided 80% power (*α* = 0.05). Patients from a School of Dentistry were selected by a nonprobabilistic method from August 2017 to April 2018. Their chief complaint was malocclusion. The inclusion criteria were subjects (males or females) on permanent dentition without prior orthodontic treatment that met the Index of Complexity, Outcome, and Need (ICON) evaluation criteria without age restrictions. Exclusion criteria were edentulous patients or those with occlusal restorations in more than 30% of teeth in addition to patients undergoing treatment with analgesic, anti-inflammatory, and/or muscle relaxant medications. Subjects with arthritic conditions or disc displacement accompanied by articular pain or dysfunction were eliminated from the control group. In total, 327 individuals were included in this study (96 cases and 231 controls). To obtain an even age distribution, the subjects were grouped into quartiles: (1) 17 years or less (Q1), (2) from 18 to 21 years (Q2), (3) from 22 to 27 years (Q3), and (4) 28 or more years old (Q4).

**Figure 1:**
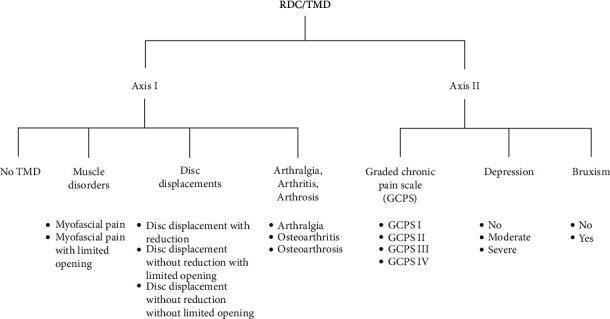
Research Diagnostic Criteria for Temporomandibular Disorders (RDC/TMD).

**Figure 2:**
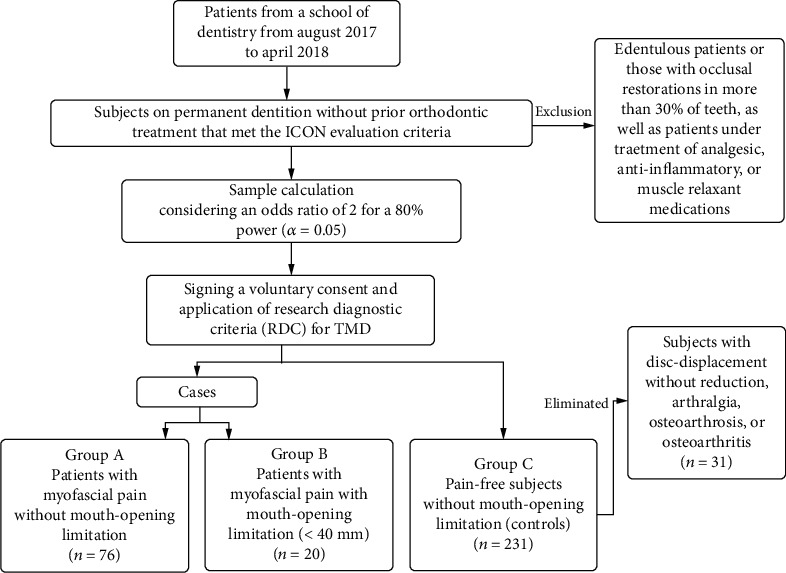
Methodology workflow chart.

The Axis I of the Research Diagnostic Criteria (RDC) for TMD [5] was used to identify cases and controls (Figure 1). To diagnose for bruxism and to estimate depression level into absent, moderate or severe the RCD/TMD Axis II was used (Figure 1). Afterwards, patients were divided into three groups: (1) group A consisted of patients with myofascial pain without mouth-opening limitation, (2) group B consisted of patients who reported myofascial pain with limited mouth-opening abilities (<40 mm) [28], and (3) a control group (group C/pain-free subjects without mouth-opening limitation) as shown in Figure 2. For the A and B groups, the grades of chronic pain were estimated based on the RDC/TMD Axis II criteria: (1) grade I: low disability with low intensity, (2) grade II: low disability with high intensity, (3) grade III: high disability with moderate limitation, and (4) grade IV: high disability with severe limitations.

**Figure 3:**
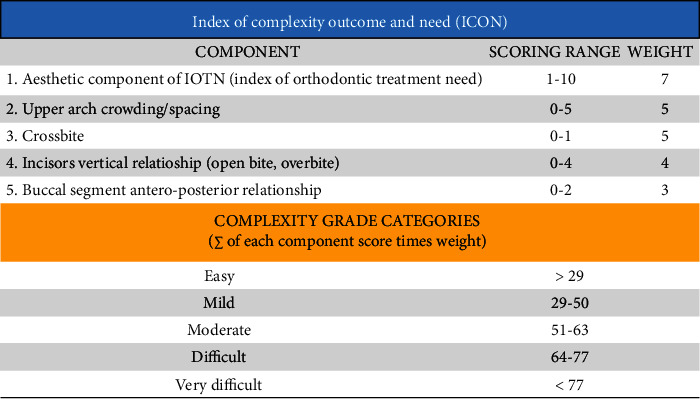
Index of Complexity, Outcome, and Need (ICON).

Afterwards, the ICON was calculated for each individual following the methods from previous publications [29, 30] (Figure 3). According to their ICON score, a patient's malocclusion was classified according to the index's categories: (1) easy (<29), (2) mild (29–50), (3) moderate (51–63), (4) difficult (64–77), and (5) very difficult (>77).

### 2.1. Method to Quantify the Error

A pilot study was performed to quantify the error of measurement for the RDC/TMD test and the ICON. A single operator measured 30 patients two times one week apart. The results were compared through the kappa coefficient of agreement (RDC/TMD: 0.84 and ICON: 0.94).

### 2.2. Statistical Analysis

The statistical analysis was performed using the SPSS software, v.20. Moreover, bivariate analysis (Chi-square test) was performed to identify associations between myofascial pain and the following variables: gender, age (divided into quartiles), malocclusion complexity (ICON), depression, and bruxism (*p* ≤ 0.05). Subsequently, a multinomial logistic regression was performed, including the variables with *p* < 0.2 in the bivariate analysis (goodness-of-fit test and Nagelkerke's *R*^2^ coefficient were estimated). Finally, the odds ratios and confidence intervals of 95% (95% CI) were measured for both tests (Chi-square and multinomial logistic regression).

## 3. Results

A total of 358 subjects were assessed; 31 were eliminated for the following reasons: 15 had disc displacement without reduction, 8 had arthralgia, 7 osteoarthrosis, and 1 osteoarthritis. The final count was of 327 subjects, of which 67% (*n* = 219) were female (24.33 ± 9.38 years), and 33% (*n* = 108) were male (23.97 ± 10.63 years). The minimum and maximum ages were 15 and 69, respectively, with an average age of 24.21 ± 9.8 years.

Concerning malocclusion complexity, 18.7% (*n* = 61) of the subjects were on the lower level (the same amount was found in the mild level), 32.1% (*n* = 105) were moderate, 22% (*n* = 72) were difficult, and 8.6% (*n* = 28) were very difficult.

Groups A and B were confirmed as 76 (79.17%) patients had myofascial pain without mouth-opening limitation and were included in group A, while 20 (20.83%) had mouth-opening limitation and were included in group B.

**Figure 4:**
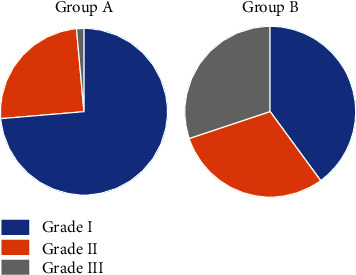
Pie charts showing the distribution of the groups A and B according to the grades of chronic pain and expressed in percentages.

Group A chronic pain distribution was 73.7% (*n* = 56) grade I, 25% (*n* = 19) grade II, and 1.3% (*n* = 1) grade III. Meanwhile, the distribution in group B was 40% (*n* = 8) grade I and 30% (*n* = 6) for grades II and III, respectively (Figure 4). A significant association was found between the groups (*p* < 0.001).

### 3.1. Bivariate Analysis

**Figure 5:**
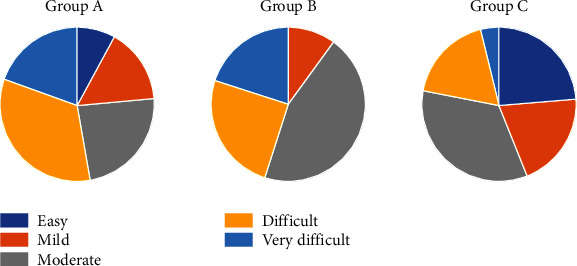
Pie charts showing the distribution of the groups according to the Index of Complexity, Outcome, and Need levels and expressed in percentages.

Statistically significant associations were found between myofascial pain levels and the variables gender, malocclusion complexity, and depression (*p* < 0.05). Results show that females were more affected in both case groups (*p* = 0.001). Regarding malocclusion complexity, most of the controls were distributed in the first three ICON levels. However, patients with myofascial pain (groups A and B) were in the last three (*p* < 0.001) (Figure 5). Concerning depression in both A and B groups, most of the patients had moderate and severe levels (*p* < 0.001). Given its respective *p* value, age was excluded from the multinomial analysis (*p* = 0.327). However, bruxism was included (*p* = 0.117).  Table 1 presents the bivariate results.

### 3.2. Multinomial Logistic Regression

The multinomial logistic regression model showed a statistically significant association of gender for both case groups (A and B) (*p* < 0.05). A significant association was noted between malocclusion complexity and myofascial pain in group A (*p* < 0.05). Moreover, an association was recorded between depression and myofascial pain in group B (*p* < 0.05).  Table 2 presents the results of the multinomial logistic regression model.

## 4. Discussion

The results suggest that gender, malocclusion complexity, and depression are associated with the presence of myofascial pain. However, their correlation is different when comparing case groups. Results showed that gender and malocclusion complexity correlated in patients without mouth-opening limitations, whereas gender and depression correlated in patients with limitations.

The relationship between TMD and gender is well documented in the literature [7]. Females are at more risk of TMD, and the results of this study agree with this fact. On the other hand, the association between malocclusion and TMD has been subject of debate in the research community for many years. Regarding myofascial pain, De Paiva et al. reported that class II or III molar malocclusion was associated with a higher prevalence in adolescents [12]. Meanwhile, Schmitter et al. found that open bite patients have an increased risk of presenting myofascial pain, and they concluded that occlusion plays a role in this presentation. Still, it is restricted to subjects with “serious alterations of (occlusal) normality” [31]. This latter statement is consistent with the results of this study in which the highest levels of complexity accompany greater odds of presenting myofascial pain. Some studies investigating malocclusion, using indexes such as ICON [20] or Peer Assessment Rating [21], concluded that the more severe the malocclusion is, the more likely TMD will occur. Since malocclusion is not an etiological factor for TMD [19], the relationship between myofascial pain without mouth-opening limitation and malocclusion complexity lies in the impact of the malocclusion on the subjects' quality of life and their psychosocial well-being [20]. Typically, psychological conditions are etiological factors for TMD development and myofascial pain [11].

However, subjects diagnosed with myofascial pain with mouth-opening limitation face more severe clinical features. Apart from the obvious pain and dysfunction, these patients also experienced depression, high levels of chronic pain, and high levels of malocclusion complexity. A study found that a significant number of patients with mouth-opening limitation are depressed [13]. It has also been proven that patients with depression are more prone to higher muscle tenderness, suggesting that patients with facial pain should be screened for psychiatric disorders [32].

As discussed previously, patients with mouth-opening limitations had the highest levels of chronic pain. While the association between chronic pain and depression has previously been reported, it is unclear if there is a strong link in their relationship. However, high levels of chronic pain are accompanied by high depression [14].

Moreover, it is proven that myofascial pain harms the quality of life, especially when accompanied by mandibular dysfunction and depression [16]. In addition, a high level of malocclusion complexity might worsen the quality of life and psychosocial well-being [22, 23], creating a more complicated scenario.

In this study, the individuals with mouth-opening limitations had the highest levels of malocclusion complexity. Thus, high levels of malocclusion complexity, chronic pain, and depression coexist in creating a complicated environment for this group of patients. However, the interactions between these variables should be a subject of further investigation.

This study has some limitations. First, as the cases were grouped, the number of patients with mouth-opening limitations might fail to satisfy a suitable group of subjects to contrast the variability in the multinomial model. Second, this study used the RDC/TMD to assess depression, but other psychological conditions not considered could affect the patients' well-being.

## 5. Conclusions

In conclusion, the results of this study demonstrate that females had greater risk of myofascial pain without mouth-opening limitation and that the highest levels of malocclusion complexity increase the odds of present this condition. Moreover, myofascial pain with mouth-opening limitation frequently coexists with depression and high levels of chronic pain.

## Figures and Tables

**Table 1:** Results of association between the presence of myofascial pain without or with mouth-opening limitation with gender, age, level of malocclusion complexity, depression, and bruxism. Chi-square test.

Variables	Group A	Group B	Group C	*p*
	% (*n* = 76)	% (*n* = 20)	% (*n* = 231)	
*Gender*				
Male	19.7% (15)	10% (2)	39.4% (91)	0.001^∗^
Female	80.3% (61)	90% (18)	60.6% (140)	
*Age*				
Q1	26.3% (20)	40% (8)	28.6% (66)	0.327
Q2	17.1% (13)	20% (4)	25.5% (59)	
Q3	31.6% (24)	10% (2)	22.5% (52)	
Q4	25% (19)	30% (6)	23.4% (54)	
*ICON*				
Easy	7.9% (6)	0% (0)	23.8% (55)	<0.001^∗^
Mild	15.8% (12)	10% (2)	20.3% (47)	
Moderate	23.7% (18)	45% (9)	33.8% (78)	
Difficult	32.9% (25)	25% (5)	18.2% (42)	
Very difficult	19.7% (15)	20% (4)	3.9% (9)	
*Depression*				
No	40.8% (31)	10% (2)	63.2% (146)	<0.001^∗^
Moderate	43.4% (33)	45% (9)	30.7% (71)	
Severe	15.8% (12)	45% (9)	6.1% (14)	
*Bruxism*				
No	39.5% (30)	55% (11)	52.8% (122)	0.117
Yes	60.5% (46)	45% (9)	47.2% (109)	

^∗^Statistically significant.

**Table 2:** Multinomial logistic regression.

Variables	Group A			Group B		
	*p*	OR	95% CI	*p*	OR	95% CI
*Gender*						
Male	0.002^∗^	0.34	0.171-0.678	0.05^∗^	0.209	0.043-1
Female^†^						
*ICON*						
Easy	<0.001^∗^	0.066	0.018-0.238			
Mild	0.001^∗^	0.138	0.045-0.423	0.055	0.143	0.02-1.044
Moderate	<0.001^∗^	0.132	0.047-0.375	0.237	0.397	0.086-1.836
Difficult	0.036^∗^	0.339	0.124-0.931	0.19	0.335	0.066-1.716
Very difficult^†^						
*Depression*						
No	0.202	0.541	0.21-1.39	<0.001^∗^	0.043	0.008-0.233
Moderate	0.235	0.57	0.225-1.442	0.013^∗^	0.235	0.075-0.739
Severe^†^						
*Bruxism*						
No	0.243	0.71	0.399-1.263	0.397	1.558	0.558-4.35
Yes^†^						

^†^Reference category. ^∗^Statistically significant; OR: odds ratio; CI: confidence interval; goodness-of-fit test Pearson: *p* = 0.907; Nagelkerke *R*^2^: 0.29.

## Data Availability

The subjects' data used to support the findings of this study are included within the article.

## Supplementary Materials

Supplementary Materials STROBE checklist.
